# Enhancing the Bioactive Properties of Sugarcane Vinegar Through *Caesalpinia sappan* Extract Supplementation: A Novel Approach for Functional Beverage Development

**DOI:** 10.3390/antiox14050590

**Published:** 2025-05-14

**Authors:** Preekamol Klanrit, Haruthairat Kitwetcharoen, Kanit Vichitphan, Sukanda Vichitphan, Sudarat Thanonkeo, Mamoru Yamada, Pornthap Thanonkeo

**Affiliations:** 1 Department of Biotechnology, Faculty of Technology, Khon Kaen University, Khon Kaen 40002, Thailand; kpreek@kku.ac.th (P.K.); kanvic@kku.ac.th (K.V.); sukanda@kku.ac.th (S.V.); 2 Fermentation Research Center for Value Added Agricultural Products (FerVAAPs), Khon Kaen University, Khon Kaen 40002, Thailand; 3 Walai Rukhavej Botanical Research Institute (WRBRI), Mahasarakham University, Maha Sarakham 44150, Thailand; kharuthairat29@gmail.com (H.K.); sudarat.t@msu.ac.th (S.T.); 4 Department of Biological Chemistry, Faculty of Agriculture, Yamaguchi University, Yamaguchi 753-8515, Japan; m-yamada@yamaguchi-u.ac.jp; 5 Research Center for Thermotolerant Microbial Resources, Yamaguchi University, Yamaguchi 753-8515, Japan

**Keywords:** acetic acid bacteria, antioxidant, bioactive compounds, *Caesalpinia sappan*, vinegar, health beverages

## Abstract

Functional vinegars have been produced from various ingredients worldwide, yet there remains a notable gap in utilizing herbal plants as complementary ingredients to sugar-based materials. This study investigates the innovative combination of *Caesalpinia sappan* extract with sugarcane juice for functional vinegar production. The results demonstrate that *C. sappan*-supplemented vinegars exhibited significantly enhanced quality parameters compared to control vinegar made from sugarcane juice alone. Specifically, the supplemented vinegars showed increased total acidity and total phenolic content (TPC), with the improvement directly proportional to the concentration of plant extract used. Gas chromatography–mass spectrometry (GC-MS) analysis revealed unique volatile organic compounds (VOCs) that were present exclusively in the *C. sappan*-supplemented vinegars but absent in the control. Most notably, the supplementation of *C. sappan* extract at concentrations of 2 and 4 g/L substantially enhanced both the antioxidant capacity and antimicrobial activity of the resulting vinegars. These biochemical improvements highlight the synergistic potential of combining sugarcane juice with *C. sappan* extract for developing novel functional vinegar beverages with enhanced bioactive properties. Our findings open new possibilities for creating value-added products that leverage traditional medicinal plants in modern functional beverages, potentially offering consumers additional health benefits beyond conventional vinegars.

## 1. Introduction

Vinegar, a liquid product generated through alcoholic and acetic fermentation, has gained increasing popularity due to growing consumer interest in functional foods and beverages. Beyond direct consumption, vinegar has diverse applications across the food and beverage, healthcare, nutraceutical, and agricultural industries [1]. Regular vinegar consumption has been associated with numerous health benefits, attributable to its rich composition of bioactive compounds including polyphenols, flavonoids, organic acids, and vitamins. These components contribute to vinegar’s biological properties, which include antioxidant, antimicrobial, antidiabetic, antiobesity, anti-inflammatory, and antihypertensive effects [2,3,4,5]. The global shift toward healthier natural alternatives has driven substantial market growth for vinegar products, with the market size reaching USD 6.54 billion in 2023 and projected to expand to approximately USD 7.98 billion by 2030 [6].

The literature reveals a diverse array of vinegar products worldwide, derived from various sources including black vinegar, rice vinegar, balsamic vinegar, white wine vinegar [3], pineapple vinegar [7,8], Corinthian currants vinegar [9], traditional sugarcane vinegar [4], and apple vinegar [5]. Furthermore, enhancing vinegar with the functional properties of herbal plants is an area of growing interest, exemplified by products such as *Gastrodia elata* vinegar [10] and wolfberry vinegar [11,12]. While these studies highlight the potential for incorporating medicinal plants, exploring different botanical supplements and substrates remains crucial. In this context, sugarcane emerges as a particularly promising base ingredient, especially given its status as one of Thailand’s most economically important agricultural crops. Thailand ranks as the world’s third-largest sugarcane producer, with substantial output (11.2 million metric tons) [13] yielding juice rich in fermentable sugars (7–16% *w*/*v*, primarily sucrose, glucose, and fructose) [14,15,16]. This composition makes sugarcane juice highly suitable for producing value-added fermented products like vinegar. Despite the existing research on functional vinegars and the suitability of sugarcane, the specific application of *Caesalpinia sappan* as a functional supplement during the fermentation of sugarcane juice for vinegar production has not, to our knowledge, been previously investigated.

The chosen botanical supplement, *C. sappan*, commonly known as sappan wood, represents a promising candidate for enhancing the functional profile of sugarcane vinegar. This tropical hardwood tree, native to Southeast Asia and the Pacific Islands, belongs to the Fabaceae family and is known by various names, including Brazilwood, Indian redwood, and Suou in different regions [17,18]. As a valuable medicinal plant, *C. sappan* is recognized for its significant antioxidant, anti-inflammatory, antimicrobial, and anticancer properties, underpinning its traditional use in treating conditions such as cough, fever, gastrointestinal problems, and skin ailments [19,20]. These health-promoting effects are largely attributed to various bioactive compounds identified in *C. sappan* wood, primarily flavonoids, phenolics, triterpenoids, steroids, alkaloids, saponins, and tannins [21], making it an ideal component for developing a value-added functional vinegar.

Therefore, this study addresses the identified research gap by developing functional vinegar using sugarcane juice supplemented with *C. sappan* wood extract. We investigate how various concentrations of the plant extract influence the physicochemical and biological properties of the resulting vinegar products. The current research findings demonstrate that the combination of sugarcane juice and *C. sappan* extract represents a promising formulation for novel functional vinegar beverages with enhanced antioxidant and antimicrobial properties, potentially expanding the functional vinegar market with a product that leverages the medicinal benefits of herbal plants.

## 2. Materials and Methods

### 2.1. Microorganisms and Chemicals

*Acetobacter pasteurianus* FPB2-3, an acetic acid bacterium isolated from fermented plant beverages, was kindly provided by Dr. Wichai Soemphol [22]. It was cultured in yeast extract–peptone–glycerol–glucose (YPGD) medium containing 0.5% yeast extract, 0.5% peptone, 0.5% glycerol, and 0.5% glucose. *Saccharomyces cerevisiae* was obtained from the Department of Biotechnology, Faculty of Technology, Khon Kaen University, and cultured in yeast extract–malt extract (YM) medium containing 0.3% yeast extract, 0.3% malt extract, 0.5% peptone, and 1.0% glucose. For both media, 2.0% agar was added to prepare solid medium.

Bacteriological grade yeast extract, malt extract, and peptone were purchased from TM medium (Titan Biotech Ltd., Delhi, India). D(+)-glucose 1-hydrate and agar were obtained from KemAus^TM^ (New South Wales, Australia), while glycerol, isopropanol, and absolute ethanol were acquired from Sigma-Aldrich (St. Louis, MO, USA).

### 2.2. Plant Materials and Their Preparation

Fresh sugarcane juice was purchased from a local market in Khon Kaen province, Thailand. The juice was filtered through four layers of cheesecloth to remove particles, and the total sugar and total phenolic content (TPC) in the juice were determined. The juice was stored at −20 °C until use. Dried *C. sappan* heartwood powder was procured from a local herbal pharmacy in the same province. The TPC and antioxidant activity of the dried powder were determined. Before using the *C. sappan* powder to produce vinegar, it underwent maceration in 95% ethanol at a 1:10 (*w*/*v*) ratio for 24 h at 30 °C. Following maceration, the ethanolic extract was filtered through Whatman No. 1 filter paper, and the liquid fraction was subsequently freeze-dried using a lyophilizer at −40 °C for 48 h to yield the *C. sappan* ethanolic extract powder. This extract powder was then used for vinegar production in the next experiments.

### 2.3. Starter Culture Preparation of Yeast and Acetic Acid Bacteria

Starter cultures of both *S. cerevisiae* and *A. pasteurianus* were prepared following similar procedures. For *S. cerevisiae*, a single colony was inoculated in 100 mL of YM medium and incubated at 30 °C with shaking at 150 rpm for 18 h. The cells were then transferred to 100 mL of fresh medium at an initial optical density (OD_600_) of 0.05 and incubated under the same conditions for 12 h. After incubation, the cells were harvested by centrifugation, washed, and resuspended in sterile distilled water to a final concentration of 10⁸ cells/mL. For *A. pasteurianus*, a single colony was inoculated in 100 mL of YPGD medium and incubated at 30 °C with shaking at 150 rpm for 18 h. Similarly, these cells were transferred to a fresh medium at an initial OD_600_ of 0.05 and incubated for 12 h. The cells were then harvested by centrifugation, washed, and resuspended in sterile distilled water to a final concentration of 10⁸ cells/mL. Both starter cultures were used for subsequent experiments.

### 2.4. Vinegar Production from Sugarcane Juice and C. sappan

Vinegar production from sugarcane juice and *C. sappan* was conducted following the protocol of Tanamool et al. [7]. The process began with the pasteurization of sugarcane juice containing 140 g/L total sugars at 65 °C for 30 min, after which it was cooled to room temperature before being transferred to 1 L fermentation jars with 650 mL working volume. Preliminary studies indicated that *C. sappan* concentrations exceeding 4.0 g/L significantly inhibited the growth of *S. cerevisiae* and *A. pasteurianus*, which informed our experimental design. Therefore, in this study, five treatment conditions with varying *C. sappan* concentrations were employed, i.e., 0 g/L (C1, control), 0.5 g/L (C2), 1.0 g/L (C3), 2.0 g/L (C4), and 4.0 g/L (C5). Each treatment received starter cultures of *S. cerevisiae* and *A. pasteurianus*, each inoculated at 5% (*v*/*v*) into the fermentation medium. Fermentation proceeded under static conditions at 30 °C, with samples collected at predetermined time intervals to monitor the biochemical and biological changes throughout the process.

### 2.5. Analytical Methods

#### 2.5.1. Volatile Organic Compounds (VOCs)

Volatile organic compounds (VOCs) in the vinegar products were determined using solid-phase microextraction (SPME) coupled with gas chromatography–mass spectrometry (GC-MS) following the methods described by Tejedor-Calvo et al. [23] and Kitwetcharoen et al. [24]. The analysis employed an Agilent 7890A GC system connected to an Agilent 7000B mass spectrometer (Agilent Technologies, Inc., Palo Alto, CA, USA). VOCs were separated using a DB-wax capillary column (60 m length, 0.25 mm diameter, 0.25 mm film thickness) with helium as the carrier gas flowing at 1 mL/min. The identification of VOCs relied on comparing sample mass spectra with the NIST MS Search Version 2.0 library database. The content of each VOC was expressed as a relative percentage area, calculated by integrating the sum of ion characteristics for each compound and determining its proportion relative to the total.

#### 2.5.2. Antioxidant Activity

The antioxidant activity of vinegar was evaluated using three complementary methods: ferric-reducing antioxidant power (FRAP) according to Phung et al. [25], 2,2-diphenyl-1-picryhydrazyl (DPPH) radical scavenging activity following Chan et al. [26], and 2,2′-azino-bis(3-ethylbenzothiazoline-6-sulfonic acid) (ABTS) radical scavenging as described by Mukherjee et al. [27]. For the FRAP assay, 50 µL of the sample was combined with 1.5 mL of FRAP reagent (containing 300 mM acetate buffer at pH 3.6, 10 mM TPTZ, and 20 mM ferric chloride) and 150 µL of distilled water. After 10 min of incubation in the dark at room temperature, absorbance was measured at 593 nm. Results were calculated using a ferrous sulfate heptahydrate standard curve and expressed as grams Fe(II)/L of the sample. For the DPPH assay, 0.2 mL of the sample was mixed with 3 mL of 0.1 mM DPPH solution and incubated in the dark at room temperature for 30 min before measuring absorbance at 517 nm. For the ABTS assay, 100 µL of the sample was added to 3.8 mL of ABTS·+ solution (prepared by combining 7 mM ABTS stock solution with 2.45 mM potassium persulfate). After 6 min of incubation at room temperature in the dark, absorbance was measured at 734 nm. Both DPPH and ABTS radical scavenging activities were calculated as the percentage of inhibition.

#### 2.5.3. Antimicrobial Activity

The antimicrobial activity of the samples against pathogenic microbes was examined using the agar-well diffusion method [25]. Six bacterial strains were employed in this study, including three gram-negative bacteria (*Escherichia coli*, *Salmonella enterica* Serovar Typhimurium, and *Enterobacter aerogenes*) and three gram-positive bacteria (*Bacillus cereus*, *Staphylococcus aureus*, and *Enterococcus faecalis*). For the antimicrobial assay, a starter culture of each bacterial strain was spread on Mueller–Hinton (MH) agar plates. Wells of 6 mm diameter were created in the agar, and 100 μL of sterile vinegar samples (filtered through a 0.22 μm sterile microfilter) were transferred into these wells. The plates were then incubated at 37 °C for 24 h. Following incubation, the inhibition zones of microbial growth were measured according to the method described by Battikh et al. [28]. Acetic acid (0.85% *w*/*v*, pH 3.0) served as a positive control, while sterile distilled water was used as a negative control.

#### 2.5.4. pH, Total Acidity, and Color

The pH of the sample was measured using a FE28 FiveEasy electronic pH meter (Mettler Toledo, Greifensee, Switzerland). Total acidity determination was conducted through titration following the protocol established by Osiripun et al. [29]. For color characterization, an Ultrascan XE SN-U3115 spectrophotometer (Hunterlab, Reston, VA, USA) was employed to quantify the vinegar products’ color attributes using the L*, a*, and b* color parameters, which represent lightness, red–green spectrum, and blue–yellow spectrum, respectively.

#### 2.5.5. Total Sugar, Total Phenolic Compound, and Ethanol Content

Total sugar content was measured using the phenol–sulfuric acid method as described by Nielsen [30]. Total phenolic content (TPC) was quantified using the Folin–Ciocalteu method following Jakubczyk et al. [31], with results expressed as milligrams of gallic acid equivalent per liter (mg GAE/L) based on a gallic acid standard curve. The ethanol concentration was determined using gas chromatography (GC-14B, Shimadzu, Kyoto, Japan) according to the protocol described by Phung et al. [25].

### 2.6. Statistical Analysis

All experiments followed a completely randomized design (CRD) with three replications. Data were analyzed using one-way analysis of variance (ANOVA) and presented as means ± standard deviations (SDs). Significant differences between treatments were determined using Tukey’s Honestly Significant Difference (HSD) test at a confidence level of 0.05 (*p* ≤ 0.05). All statistical analyses were performed using SPSS version 28.0 (IBM SPSS Statistics, IBM Corporation, Armonk, NY, USA). Principal component analysis (PCA) was conducted to highlight the main contributors to the variance among treatments.

## 3. Results

### 3.1. Characteristics of Raw Materials

The sugar content in the raw material is a key component that directly influences ethanol and acid production during vinegar fermentation. In this study, sugarcane juice was selected as the carbon source for microbial growth and metabolic activity due to its high sugar content, particularly sucrose, glucose, and fructose. Previous research indicates that the total sugar content in sugarcane juice typically ranges from 7 to 16% (*w*/*v*), varying based on sugarcane variety, geological plantation location, and climate conditions [14,15,16]. Our analysis demonstrated that the sugarcane juice used here contained 139.78 g/L (14% *w*/*v*) of total sugar, aligning with these previously reported ranges. This concentration is particularly suitable for vinegar production, as Tanamool et al. [7] established that a sugar concentration of 140 g/L yields optimal vinegar products in terms of physicochemical properties. Consequently, we used the sugarcane juice at its natural sugar concentration (139.78 g/L) in subsequent experiments without additional sugar supplementation.

Sugarcane juice is valued not only for its sugar but also for its content of health-beneficial compounds, especially phenolics, which demonstrate diverse biological activities such as antioxidant, anti-inflammatory, antimicrobial, anticancer, and hepatoprotective effects [32]. A variety of specific phenolic compounds, including apigenin, luteolin, tricin, caffeic acid, hydroxycinnamic acids, sinapic acid, benzoic acid, ferulic acid, quinic acid, chlorogenic acid, *p*-coumaric acid, and kaempferol, among others, have been previously identified in sugarcane juice [33,34,35]. Reflecting this phenolic composition, the TPC measured in our sugarcane juice sample was 0.21 g GAE/L. This finding is comparable to previous reports, although variations exist: it was slightly higher than values reported by Duarte-Almeida et al. [33] (0.16 g chlorogenic acid equivalents/L), Chen et al. [34] (0.02–0.03 g/L), and Rodrigues et al. [35] (0.03 g/L), but lower than that found by Eggleston [36] (0.42–0.57 g GAE/L). These observed differences in the TPC are commonly attributed to factors like plant variety, cultivation conditions, harvesting processes, and analytical techniques.

*C. sappan* is known to contain various bioactive compounds, including phenolics, which contribute to its numerous biological and pharmacological properties, such as antioxidant, antimicrobial, and anti-inflammatory activities, among others [21]. Analysis of the *C. sappan* extract revealed a phenolic content of 44.69 mg GAE/g dry weight (DW) and DPPH antioxidant activity of 44.40 mg TE/g DW, with an IC_50_ of 140 ppm (Table 1). The phenolic content detected in this study was significantly higher than the values reported by Arsiningtyas [37] (4.43–8.85 mg GAE/g DW) but lower than that reported by Septiyani et al. [38] (213.12 mg GAE/g DW). Interestingly, the plant sample in this study exhibited a higher IC_50_ value compared to previous reports [37,38]. These variations in phenolic content and biological properties can likely be attributed to differences in the plant age, growing conditions, and extraction processes used across studies.

### 3.2. Physicochemical Characteristics of Vinegar Produced from Fusions of Sugarcane Juice and C. sappan Extract

**Figure 1:**
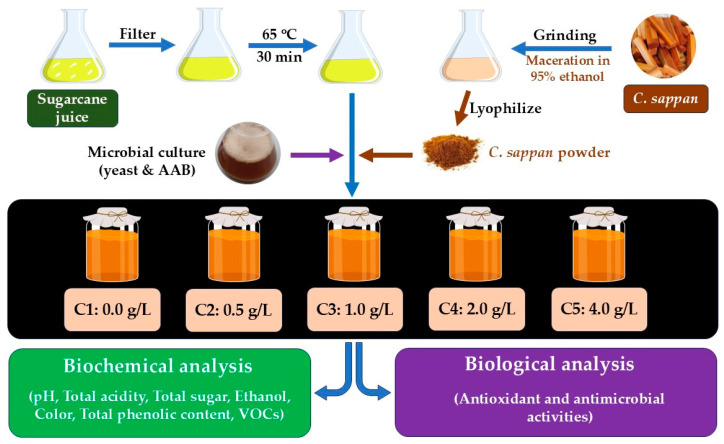
A flow diagram for the production of sugarcane juice vinegar supplemented with various concentrations of *C. sappan* extract (C1, 0 g/L; C2, 0.5 g/L; C3, 1.0 g/L; C4, 2.0 g/L; and C5, 4.0 g/L).

The overall fermentation process of sugarcane juice vinegar supplemented with *C. sappan* extract is summarized in Figure 1. During vinegar production, fermentation parameters, including pH, total acidity, total sugar content, TPC, VOCs, and color, were assessed. Furthermore, the biological properties of the vinegar products were also determined, and the results are as follows:

#### 3.2.1. pH and Total Acidity During Fermentation

The pH of the fermentation medium significantly influences microbial growth and enzyme activity in microbial cells. Additionally, pH contributes to the stability of beneficial compounds in the final products [39,40]. The literature indicates that alcoholic beverages such as beer, vinegar, and kombucha require an initial pH of approximately 4.5–5.5 [7,24,41]. For this reason, the initial pH of 5.5, the original pH of the sugarcane juice, was selected for vinegar production in this study.

**Figure 2:**
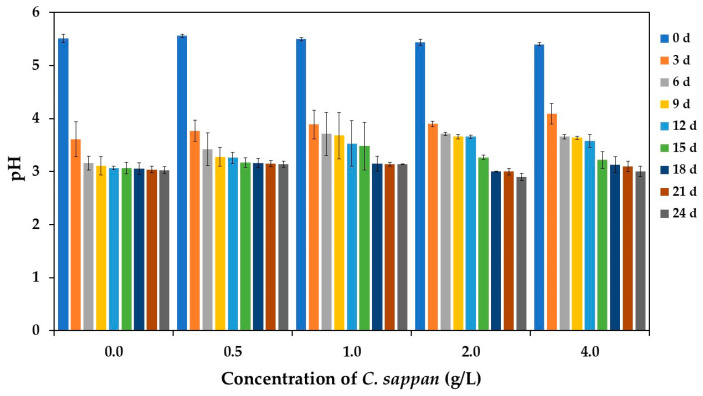
pH changes during vinegar production from sugarcane juice supplemented with *C. sappan* extract at different concentrations for 24 days.

The fermentation pH profiles were significantly influenced by the concentration of added *C. sappan* extract (Figure 2). Although the pH of all treatments dropped sharply during the first 3 days, the control group without *C. sappan* supplementation exhibited the fastest initial pH drop within this period. In contrast, increasing concentrations of *C. sappan* extract progressively slowed this initial rate of acidification, likely due to the bioactivity of its constituents (e.g., phenolics and flavonoids [21]) on the fermentative microbes. Importantly, despite these varied initial kinetics, all fermentations ultimately reached a similar final pH range of 2.90 to 3.14. This final pH range is consistent with established vinegar products like apple vinegar (2.84–3.82) [5] and aligns with general fermentation trends reported previously [8,42]. However, the novel observation in this study is the clear concentration-dependent effect of *C. sappan* extract in modulating the initial fermentation dynamics, specifically the rate of pH decline.

**Figure 3:**
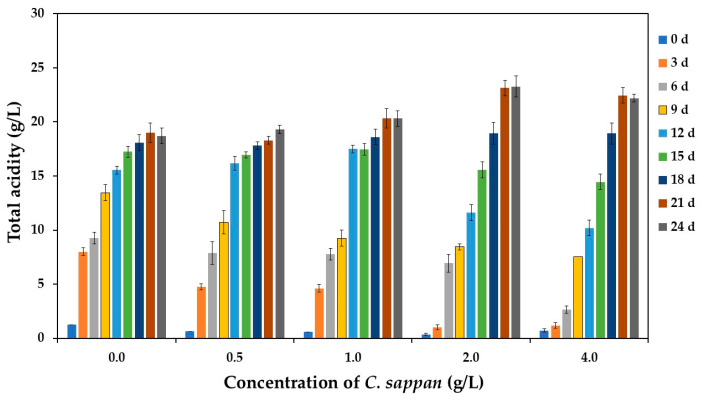
Total acidity content during vinegar production from sugarcane juice supplemented with *C. sappan* extract at different concentrations for 24 days.

Analysis of total acidity accumulation revealed a concentration-dependent effect of *C. sappan* extract (Figure 3). Initially, acidity increased most rapidly in the control group (without *C. sappan* addition), whereas supplementation of *C. sappan* extract resulted in a slower initial rate of acid production. This aligns with the observed pH trends (Figure 2) and suggests modulation of microbial growth or metabolic activity by extract constituents like phenolics or flavonoids during the early fermentation phase.

Despite these differences in initial kinetics, all fermentations reached substantial final total acidity levels, ranging from 18.71 to 23.27 g/L. This final acidity range is notably within the upper end reported for commercial apple vinegar (2.80–25.60 g/L) [5], indicating successful acidification comparable to established products and distinct from the lower levels reported for some other fruit vinegars [8]. Interestingly, the highest final total acidity values were achieved in the groups supplemented with 2.0 and 4.0 g/L *C. sappan* extract. This finding, occurring despite the slower initial microbial production rate, suggests a potential contribution of organic acids inherent to the sappan wood (e.g., chlorogenic, caffeic, gallic acids [43]) being released into the medium during fermentation. Thus, the addition of *C. sappan* extract appears to modulate the fermentation rate while potentially enhancing the final acidity through direct contribution from the extract itself, particularly at higher concentrations.

#### 3.2.2. Total Sugar During Fermentation, Ethanol Content, and Color of the Final Products

During fermentation, yeast utilizes sugar in the fermentation medium as a primary carbon source for growth and metabolism, simultaneously converting these sugars into ethanol and other beneficial compounds, including organic acids, amino acids, and vitamins [44]. The ethanol generated during this initial fermentation phase subsequently serves as a precursor for acetic acid synthesis, which is catalyzed by acetic acid bacteria through the sequential action of two membrane-bound enzymes: alcohol dehydrogenase and acetaldehyde dehydrogenase [45].

**Figure 4:**
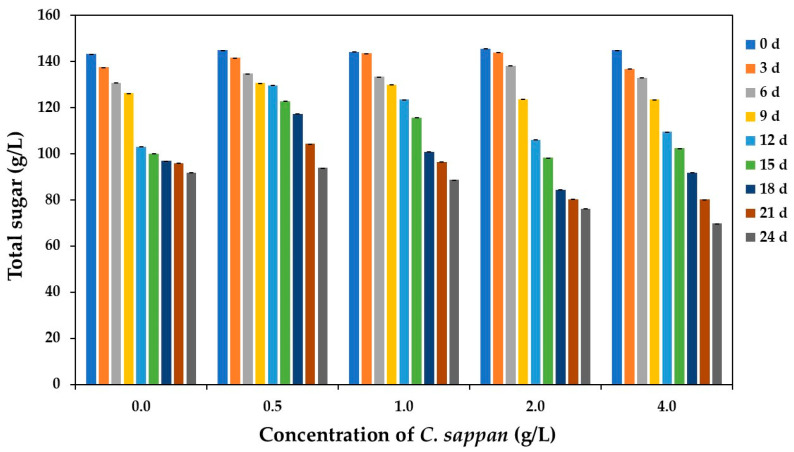
Total sugar content during vinegar production from sugarcane juice supplemented with *C. sappan* extract at different concentrations for 24 days.

As illustrated in Figure 4, all treatments exhibited a progressive decrease in sugar concentration throughout the fermentation period. Notably, the C1 treatment demonstrated a particularly rapid decline in sugar content at day 12 of fermentation. Upon completion of the 24-day fermentation cycle, the final sugar concentrations ranged from 69.69 to 93.84 g/L across all treatments. These measurements indicate that approximately 32.97–50.22% of the initial sugar content was metabolized during the 24-day fermentation period.

Interestingly, treatments supplemented with *C. sappan* extract at concentrations of 1.0, 2.0, and 4.0 g/L exhibited significantly higher sugar utilization efficiency compared to other treatments. This enhanced metabolic activity can likely be attributed to the diverse array of beneficial compounds present in *C. sappan* extract, particularly trace elements that serve as enzyme cofactors. Previous research has established that *C. sappan* contains not only bioactive compounds such as flavonoids, phenolic acids, and anthraquinones but also essential trace elements, including magnesium, zinc, and copper [46]. These micronutrients function as critical cofactors for multiple enzymes involved in the metabolic and biosynthetic pathways of ethanol production and subsequent acetic acid formation. Furthermore, the flavonoids and phenolic compounds in *C. sappan* may create favorable conditions for microbial growth by modulating oxidative stress in the fermentation medium, thereby enhancing the overall fermentation efficiency.

Previous studies have documented substantial variation in ethanol content across vinegar and vinegar products, with concentrations ranging from 0.12% to 10.71% (*v*/*v*). This variability primarily originates from differences in raw materials, microbial strains, and fermentation conditions employed during production [7,8,9]. In the present study, the sugarcane juice vinegar exhibited notably low final ethanol concentrations, measuring between 0.05% and 0.18% (*v*/*v*) (Table 2). These values align with the lower end of the ethanol range previously reported for vinegar produced from pineapple [7]. The low ethanol content can be attributed to efficient acetification during the second fermentation stage, where acetic acid bacteria effectively converted ethanol to acetic acid, resulting in minimal residual alcohol.

Based on these measurements, the vinegar produced in this study qualifies as a low-alcoholic beverage according to international standards, which typically classify products with less than 0.5% (*v*/*v*) ethanol as non-alcoholic or low-alcohol alternatives [47,48,49]. This characteristic enhances the product’s versatility and market potential as a functional beverage suitable for consumers seeking the health benefits of vinegar without significant alcohol content. Additionally, these low ethanol levels make the product appropriate for various dietary restrictions and cultural preferences that limit alcohol consumption while still delivering the probiotic benefits and bioactive compounds associated with naturally fermented beverages.

Analysis of the final products revealed distinct color variations among the vinegar samples. The control product made from sugarcane juice without *C. sappan* extract supplementation displayed a lighter yellow hue characteristic of natural sugarcane juice, as evidenced by its L*, a*, and b* values. In contrast, samples supplemented with *C. sappan* extract exhibited progressively deeper reddish-brown coloration as the concentration of the wood extract increased. This color transformation can be primarily attributed to brazilin, the principal bioactive compound in *C. sappan* extract. Brazilin functions as a natural red pigment responsible for the distinctive crimson color of the heartwood. Beyond its chromatic properties, brazilin possesses remarkable biological activities, including potent antioxidant, anti-inflammatory, and anticancer properties [50]. These bioactive characteristics potentially enhance the functional properties of the resulting vinegar beyond mere aesthetic appeal.

The application of *C. sappan* extract as a natural colorant offers significant advantages over synthetic alternatives. Previous research has demonstrated that *C. sappan* extract can effectively enhance the visual appeal of beverages and confectionery products without negatively impacting their sensory profiles—preserving their original taste and aroma characteristics [21]. This dual functionality as both a bioactive ingredient and natural colorant aligns with growing consumer preference for clean-label products free from artificial additives. Furthermore, the color stability of *C. sappan*-derived pigments across various pH levels makes it particularly suitable for acidic products like vinegar. The pronounced reddish-brown hue may also serve as a visual indicator of the product’s enhanced bioactive content, potentially increasing consumer perception of its health benefits and premium quality.

#### 3.2.3. Volatile Organic Compounds (VOCs) in Vinegar Products

Vinegar products contain numerous VOCs that contribute significantly to their flavor, aroma, and organoleptic properties, while also influencing their biological activities. Research has identified several VOC categories in these products, including esters, alcohols, carboxylic acids, aldehydes, ketones, acetals, lactones, terpenes, volatile phenols, pyrazines, and benzenes [7,8,9]. The composition and concentration of these VOCs vary considerably based on factors such as the raw materials used, production processes, and microbial strains used during fermentation. Multiple studies have documented the diversity of VOCs in various vinegar products. Tanamool et al. [7] identified 17 VOCs in pineapple vinegar, categorized as esters (4), alcohols (4), carboxylic acids (5), aldehydes (2), ketones (1), and benzenes (1). In a more extensive analysis, Plioni et al. [9] detected 140 VOCs in Corinthian currants vinegar, including carbonyl compounds (37), esters (26), alcohols (25), organic acids (13), terpenes (13), hydrocarbons (8), lactones (7), and other compounds, primarily pyrazines (11). In contrast, Boondaeng et al. [8] reported just eight VOCs in their pineapple vinegar sample, comprising esters (4), alcohols (3), and carboxylic acid (1).

**Figure 5:**
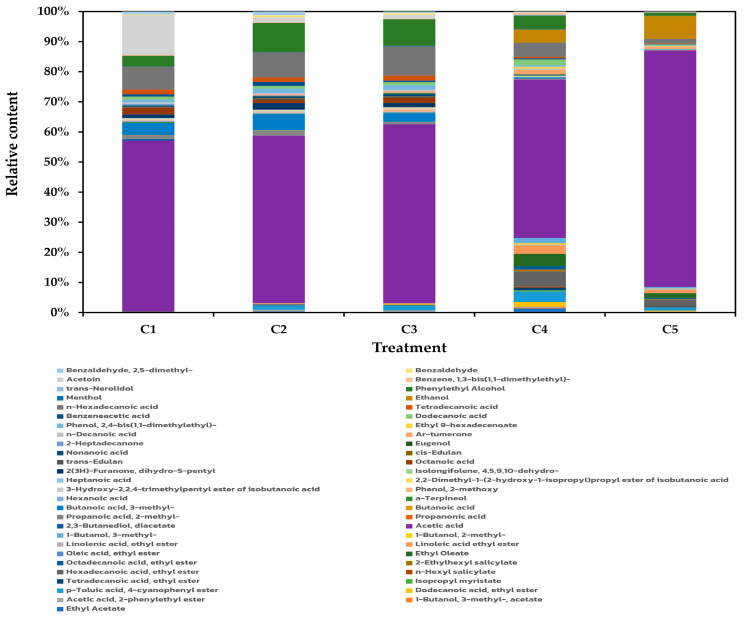
Volatile organic compounds (VOCs) detected in vinegar produced from sugarcane juice supplemented with *C. sappan* extract at 0 g/L (C1), 0.5 g/L (C2), 1.0 g/L (C3), 2.0 g/L (C4), and 4.0 g/L (C5).

In our current study, GC-MS analysis revealed 53 distinct VOCs across vinegar products, consisting of carboxylic acids (30), esters (15), alcohols (4), aldehydes (2), benzenes (1), and ketones (1), as illustrated in Figure 5 and Supplementary Materials Table S1. Among the carboxylic acids, acetic acid, n-hexadecanoic acid, and 3-methyl butanoic acid were most prevalent. The predominant esters were ethyl acetate and *p*-toluic acid,4-cyanophenyl ester, while ethanol and phenylethyl alcohol represented the major alcohols. Benzaldehyde and 2,5-dimethyl benzaldehyde constituted the principal aldehydes, with acetoin emerging as the primary ketone.

These VOCs significantly shape the sensory profile of vinegar products, each contributing distinctive aromatic and flavor characteristics. Ethyl acetate, for example, contributes appealing fruity and grape-like notes that enhance the overall bouquet, while 3-methyl butanoic acid (isovaleric acid) adds complementary fruity characteristics that enrich the flavor complexity. The *p*-toluic acid,4-cyanophenyl ester imparts delicate fruity and floral elements that provide depth to the aroma profile, and phenylethyl alcohol delivers elegant rose-like fragrances that elevate the sensory experience. 3-Methyl-butanoic acid distributes a unique floral-sweaty odor that, despite its description, balances the overall aroma in appropriate concentrations, while the benzaldehyde compounds create pleasant almond-like, fruity scents that round out the flavor profile [9,24,51]. Acetoin deserves special mention as it delivers buttery and creamy overtones and serves as an important marker of biological origin in vinegar, with its formation relating directly to acetic acid bacterial metabolism during the acetification process [9]. The presence of these aromatic compounds is not unique to this vinegar, as several of these VOCs can also be found in other vinegar varieties, such as pineapple vinegar, wine vinegar, and blueberry vinegar, suggesting common metabolic pathways in the fermentation processes across different substrate materials [7,8,52,53]. The specific concentration and combination of these compounds, however, create the distinctive sensory signature that differentiates *C. sappan*-supplemented vinegar from other vinegar products.

A key observation in this study was the significant alteration of the VOC profile in sugarcane vinegar following the addition of *C. sappan* extract. Notably, a total of 21 VOCs were detected exclusively in the vinegars supplemented with *C. sappan* and were absent in the control samples prepared from sugarcane juice alone. These distinguishing compounds include ethyl acetate, isoamyl acetate (1-butanol, 3-methyl-acetate), dodecanoic acid ethyl ester, isopropyl myristate, tetradecanoic acid ethyl ester, n-hexyl salicylate, 2-ethylhexyl salicylate, octadecanoic acid ethyl ester, ethyl oleate, oleic acid ethyl ester, linoleic acid ethyl ester, linolenic acid ethyl ester, ethyl 9-hexadecanoate, *p*-toluic acid, 4-cyanophenyl ester, 1-butanol, 2-methyl and 1-butanol, 3-methyl (isoamyl alcohol), propanoic acid, isolongifolene, 4,5,9,10-dehydro, eugenol, Ar-turmerone, and trans-nerolidol. The presence of these unique VOCs strongly suggests that *C. sappan* significantly influences the aroma profile of the final vinegar product. These compounds could potentially originate from two primary sources: (i) direct contribution from the *C. sappan* plant material itself or components within the sugarcane juice whose volatility or formation is influenced by the extract or (ii) generation through microbial metabolic activities during the fermentation process acting upon precursors from either the sugarcane juice or the added extract.

Based on their chemical nature, some distinctions can be hypothesized. Many of the identified esters (e.g., ethyl acetate, isoamyl acetate, various ethyl esters of fatty acids) and higher alcohols (e.g., 2-methyl-1-butanol, 3-methyl-1-butanol) are well-established byproducts of yeast and bacterial metabolism, typically formed via sugar, amino acid, and lipid pathways during fermentation. Their increased presence or exclusive detection in the supplemented vinegars could indicate that *C. sappan* constituents either provide additional precursors or stimulate specific microbial metabolic routes. Conversely, compounds such as eugenol, Ar-turmerone, trans-nerolidol, and sesquiterpene (isolongifolene) are often characteristic secondary metabolites found in plant tissues. It is plausible that these VOCs, or their non-volatile precursors, were directly introduced via the *C. sappan* extract. Previous studies have documented that *C. sappan* is rich in diverse bioactive compounds, including flavonoids, phenolic acids, terpenoids, anthraquinones, steroids, alkaloids, and others [21], which could serve as direct sources of VOCs or be biotransformed by fermentative microbes into volatile derivatives, further contributing to the unique chemical signature of the supplemented vinegars.

However, it is important to acknowledge a limitation of the current study: the specific VOC profiles of the initial raw materials (sugarcane juice and the *C. sappan* extract utilized) were not determined prior to fermentation. Without this baseline chemical analysis, it is not possible to definitively ascertain whether a specific VOC detected exclusively in the supplemented vinegars directly contributed by the *C. sappan* extract or if it was synthesized de novo or biotransformed from precursors during the fermentation process. Consequently, while the data clearly demonstrate the profound impact of *C. sappan* addition on the vinegar’s aromatic complexity, precisely attributing the origin of each unique VOC requires further investigation. Future studies incorporating comprehensive VOC analysis of the raw materials, alongside tracking VOC evolution throughout the fermentation stages, would be invaluable for rigorously distinguishing between directly contributed and microbially generated compounds, thereby fully elucidating the mechanisms behind the observed aroma enhancement.

Beyond their contributions to flavor and aroma profiles, several of the VOCs detected in the *C. sappan*-supplemented vinegar products possess significant health-promoting properties. Acetic acid demonstrates multifaceted biological activities, functioning as an antioxidant, anti-inflammatory, and antimicrobial compound [54,55]. This primary component of vinegar has been associated with improved glucose control and weight management in multiple studies. Menthol has been extensively documented to possess anti-inflammatory, anticholinesterase, and antibacterial activities [56,57]. Its cooling sensation and analgesic properties make it valuable for both culinary and therapeutic applications. Linoleic acid ethyl ester exhibits notable antidiabetic and antibacterial activities. As a derivative of an essential omega-6 fatty acid, it may contribute to maintaining proper cell membrane function and inflammatory regulation [58,59].

Additionally, Ar-turmerone possesses a remarkable range of biological activities, including anti-inflammatory, anti-immunomodulatory, antifungal, and anticancer properties [60,61,62]. This compound, also found in turmeric, has shown particular promise in neurological applications due to its ability to cross the blood–brain barrier. Eugenol, another identified compound, demonstrates antimicrobial, anti-inflammatory, and antioxidant properties, making it valuable in both food preservation and therapeutic contexts [63]. The synergistic interactions between these bioactive VOCs may enhance the overall health benefits of *C. sappan*-supplemented vinegar compared to conventional products, suggesting potential applications in functional foods and nutraceuticals. Further research is warranted to elucidate the specific mechanisms through which these compounds exert their beneficial effects and to optimize fermentation conditions for maximizing their production in *C. sappan*-supplemented vinegar.

#### 3.2.4. Total Phenolic Content (TPC) in the Vinegar Products

The phenolic content in vinegar primarily originates from the raw materials used in production, with concentrations varying based on the type and quantity of ingredients, as well as fermentation conditions—similar patterns are observed in other functional beverages such as wine and kombucha [24,64,65]. Research has demonstrated that beneficial phenolic compounds can also be generated through microbial metabolic activity during vinegar fermentation, though their concentrations fluctuate depending on the raw materials, supplementary ingredients, microbial community composition, and specific fermentation parameters. Polyphenols are particularly valuable compounds due to their numerous health benefits, including antioxidant, antimicrobial, anti-inflammatory, and anticancer properties [43,66,67]. These health-promoting attributes make monitoring TPC an essential aspect for quality control for vinegar products, providing manufacturers with a reliable metric for assessing potential functional benefits.

**Figure 6:**
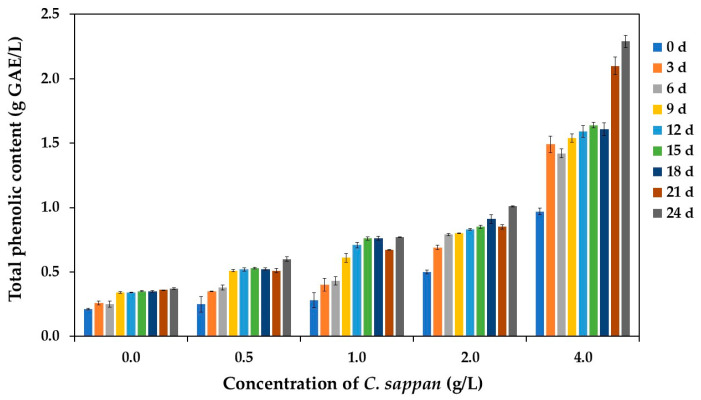
Total phenolic content (TPC) in vinegar produced from sugarcane juice supplemented with *C. sappan* extract at different concentrations for 24 days.

Based on the results presented in Figure 6, the control sample (without *C. sappan* extract) exhibited the lowest TPC at the beginning of fermentation (0 h), likely due to the inherently low phenolic content of sugarcane juice. In contrast, the initial TPC values in samples supplemented with *C. sappan* extract (0.5, 1.0, 2.0, and 4.0 g/L) were progressively higher, demonstrating a dose-dependent release of phenolic compounds from the added extract into the vinegar product.

Furthermore, monitoring TPC during fermentation revealed an increasing trend in all experimental groups, though the magnitude of this increase was less pronounced in the control treatment compared to the *C. sappan*-supplemented groups. Notably, the 4 g/L *C. sappan* treatment consistently displayed the highest TPC values throughout the fermentation periods. This progressive increase in TPC during fermentation, particularly in the supplemented groups, likely arises from several mechanisms. Firstly, the ethanol and acetic acid produced during fermentation may enhance the dissolution of polyphenols from the plant extract. Secondly, microbial enzymes could release phenolics that were initially bound within the plant matrix. Thirdly, biosynthesis of phenolic compounds by the fermenting microorganisms might also contribute. Supporting these explanations, *C. sappan* is known to contain numerous polyphenolic compounds like chlorogenic acid, caffeic acid, and gallic acid [43]. Additionally, the enhanced dissolution under acidic conditions is consistent with findings by Asfar et al. [68], who showed that low pH significantly improves polyphenol extraction and solubility from *C. sappan* wood, mirroring the acidic environment of vinegar fermentation. Overall, these findings highlight *C. sappan* extract as an effective TPC-enhancing agent in vinegar, comparable to results seen with other supplements like red dragon fruit [8].

#### 3.2.5. Antioxidant Activity of Vinegar Products

**Figure 7:**
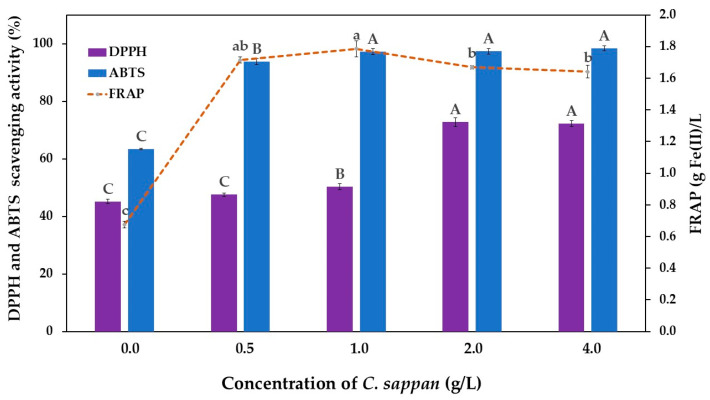
Antioxidant activity of vinegar produced from sugarcane juice supplemented with *C. sappan* extract at different concentrations for 24 days. Bars following different letters (uppercase for DPPH and ABTS, and lowercase letters for FRAP) in each parameter differed significantly based on Tukey HSD analysis at *p* ≤ 0.05.

The bioactive compounds in vinegar, whether derived from raw materials or produced through metabolic pathways during biological fermentation, demonstrate a strong correlation with antioxidant properties. Liu et al. [69] established that the antioxidant activity of vinegar products is closely linked to their phenolic and organic acid contents, with the level of activity varying based on raw materials and detection methodologies. As a specific example, Ozturk et al. [70] documented DPPH free radical scavenging activity in apple vinegars ranging from 0.53% to 65.12%, and Hasan et al. [71] reported the values ranging from 19.11% to 86.77%. In the current study, all vinegar products exhibited substantial antioxidant capabilities, as illustrated in Figure 7. Notably, vinegar products supplemented with *C. sappan* demonstrated enhanced antioxidant capacities compared to the control sample without supplementation, as measured by DPPH, ABTS, and FRAP assays. The *C. sappan*-supplemented products showed DPPH and ABTS radical scavenging activities of 47.56–72.75% and 93.70–98.34%, respectively, significantly exceeding the control sample values of 45.14% and 63.34%. Similarly, the FRAP activity values for *C. sappan*-supplemented samples ranged from 1.64 to 1.79 g Fe(II)/L, more than double that of the control sample (0.68 g Fe(II)/L). This enhanced antioxidant profile correlates strongly with the higher total acidity (measured as acetic acid) observed in Figure 3 and the elevated TPC shown in Figure 6. The results obtained in this study aligned with those reported by Boondaeng et al. [8] and Abdali et al. [5].

Beyond the organic acids and phenolic compounds identified in the vinegar products, the increased antioxidant activity in sugarcane-juice-based vinegar supplemented with *C. sappan* extract may be attributed to additional bioactive compounds from this plant. Yohana et al. [72] highlighted that *C. sappan* contains potent antioxidant compounds, including brazilin, protosappanin, and sappanone, which may contribute to the overall antioxidant effect. These compounds have been shown in other studies to possess anti-inflammatory, antimicrobial, and anti-cancer properties as well [21], potentially enhancing the functional benefits of *C. sappan*-supplemented vinegar. Further investigation is warranted to quantify and characterize these specific compounds in the products and to determine their bioavailability and stability during storage.

#### 3.2.6. Antimicrobial Activity of Vinegar Products

Vinegar demonstrates significant antimicrobial activity against a variety of pathogenic bacteria and yeasts. This biological property is directly linked to its rich profile of bioactive compounds, including phenolics, flavonoids, organic acids, alcohols, and volatile organic compounds (VOCs). Recent research by Abdali et al. [5] has highlighted the considerable potential of apple-derived vinegar in inhibiting the growth of both gram-negative bacteria (*E. coli*) and gram-positive bacteria (*B. subtilis* and *S. aureus*), as well as yeasts such as *Candida albicans*—microorganisms notorious for developing multidrug resistance and causing frequent infections in both community and hospital settings.

Our current study evaluated the antimicrobial activity of vinegar produced from sugarcane juice supplemented with *C. sappan* extract against various bacteria. As detailed in Table 3, all vinegar formulations exhibited antimicrobial properties, though with varying degrees of effectiveness. Control products without *C. sappan* extract showed the lowest inhibition capacity, with inhibition zones measuring only 6.6–7.6 mm for gram-negative bacteria and 7.3–8.4 mm for gram-positive bacteria. Notably, vinegar products supplemented with *C. sappan* extract demonstrated significantly enhanced antimicrobial activity compared to the controls, with inhibition effectiveness increasing proportionally to the concentration of plant extract. The most potent inhibition was observed in vinegar containing 4.0 g/L of *C. sappan* extract, achieving antimicrobial efficacy comparable to the positive control of acetic acid (pH 3.0). Acetic acid functions as a powerful antimicrobial agent by penetrating microbial membranes, reducing intracellular pH through proton release into the cytoplasm, and ultimately causing microbial death [73]. These results align with previous findings reported by Osuala et al. [74] and Abdali et al. [5].

The enhanced antimicrobial properties of *C. sappan*-supplemented vinegar can be attributed not only to elevated levels of organic acids, particularly acetic acid, but also to additional beneficial compounds released from the plant during fermentation. Srinivasan et al. [75] reported that *C. sappan* wood contains substantial amounts of flavonoids and tannins that effectively inhibit the growth of various bacteria, including *S. typhimurium*, *E. coli*, and *S. aureus*. Furthermore, recent research by Vij et al. [21] has identified numerous antimicrobial compounds in *C. sappan*, including brazilin, brazilein, hematoxylin, protosappanin, sappanone A, chlorogenic acid, caffeic acid, gallic acid, lupeol, β-amyrin, and cycloartenol.

Several VOCs detected in the *C. sappan*-supplemented vinegars may also contribute to antimicrobial activity, including menthol, linoleic acid ethyl ester, Ar-turmeone, and eugenol [57,59,62,63]. These compounds likely interact with bacterial cells, causing membrane damage, metabolic dysfunction, and cellular disruption that impede microbial growth [76]. It is particularly noteworthy that, consistent with previous findings by Yagnik et al. [77], El-Sayed et al. [78], and Abdali et al. [5], gram-positive bacteria exhibited greater sensitivity to the vinegar products than gram-negative bacteria. This differential sensitivity can be attributed to structural differences in bacterial cell walls—gram-positive bacteria possess a more permeable cell wall structure, whereas gram-negative bacteria are protected by two membrane layers that function as a barrier against antimicrobial compounds [79].

#### 3.2.7. Principal Component Analysis (PCA)

**Figure 8:**
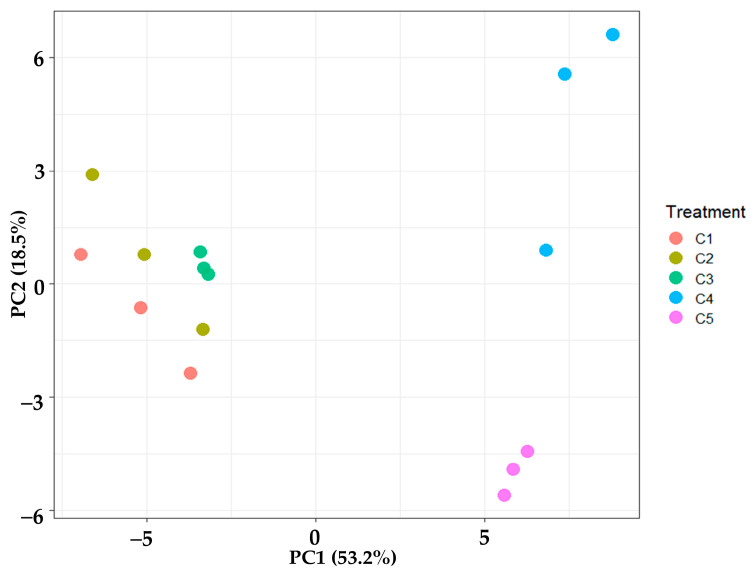
Principal component analysis (PCA), based on biochemical and biological properties, of vinegar produced from sugarcane juice supplemented with *C. sappan* extract at 0 g/L (C1), 0.5 g/L (C2), 1.0 g/L (C3), 2.0 g/L (C4), and 4.0 g/L (C5).

Principal component analysis (PCA) was performed to investigate the multivariate differences among the five treatments based on their VOCs, total acidity, total sugar, ethanol content, TPC, antioxidant activities (DPPH, ABTS, FRAP), and antimicrobial activity. The results are summarized in Figure 8. The first two principal components (PC1 and PC2) accounted for 53.2% and 18.5% of the total variance, respectively, cumulatively explaining 71.7% of the data variability. The PCA score plot (Figure 8) revealed distinct groupings: treatments C1, C2, and C3 formed a tight cluster, indicating similar profiles for the measured parameters, while the tight clustering of replicates within each treatment confirmed experimental consistency. In contrast, treatments C4 and C5 were clearly separated from both the C1–C3 group and each other. This distinct positioning suggests that the specific treatments applied to C4 and C5 induced substantial alterations to their overall chemical and biological characteristics compared to the other samples.

Further examination of the loading plots provided insights into these separations. Variables such as TPC and antioxidant activity (DPPH, ABTS, FRAP assays) contributed most strongly to the separation along PC1, which primarily distinguished C4 and C5. PC2, on the other hand, was mainly influenced by parameters like antimicrobial activity and total sugar content, contributing to the differentiation between C4 and C5 themselves, as well as from the C1–C3 cluster. Therefore, the clear separation of C4 and C5 in the PCA, driven largely by their TPC, antioxidant capacity, and antimicrobial activity, underscores the effectiveness of these treatments in enhancing specific functional properties. These findings align with their unique compositional profiles, supporting the potential of C4 and C5 as formulations with superior health-promoting characteristics compared to the control and other treatments.

This study demonstrated that the vinegar produced from sugarcane juice supplemented with *C. sappan* extract exhibited promising quality characteristics, including optimal pH value, total acidity, total sugar, ethanol content, VOCs, and TPC, along with significant biological properties evidenced by its antioxidant and antimicrobial capacities. While these findings are encouraging, a comprehensive understanding requires further characterization, particularly concerning its sensory attributes, which are critical for a functional beverage. A notable limitation of this research is indeed the absence of sensory evaluation, restricting our ability to fully correlate the desirable physicochemical and biological attributes with organoleptic properties. Therefore, future research incorporating comprehensive sensory analysis is essential. This will provide crucial insights into consumer perception and acceptance.

To further support and interpret future sensory findings, building upon the VOC profiling conducted in this study is also warranted. While the current analysis identified key volatile compounds using relative percentages, future investigations should focus on the absolute quantification of major constituents (such as dominant acids and esters). Determining the precise concentrations of these compounds will enable a more thorough understanding of their individual contributions to the specific sensory characteristics (especially aroma) and potential functional attributes of the sugarcane vinegar, clarifying the impact of *C. sappan* extract supplementation. Ultimately, integrating quantitative VOC data with comprehensive sensory results and the existing physicochemical and biological findings will offer a holistic understanding of these novel vinegar formulations. Such an integrated analysis will provide valuable scientific insights and practical guidance for manufacturers seeking to optimize ingredient combinations that satisfy consumer preferences while maintaining the beneficial properties of the final products.

## 4. Conclusions

This study demonstrates that combining sugarcane juice with *C. sappan* extract provides an excellent basis for producing functional vinegar beverages. While sugarcane juice provides the essential substrate for efficient alcoholic and acetic acid fermentation, the key innovation lies in the supplementation with *C. sappan*. The extract contributes significant phenolic compounds (44.69 mg GAE/g DW), known for their health benefits, leading to substantial enhancements in the final product. Specifically, incorporating *C. sappan* yielded notable improvements in the vinegar’s TPC and VOC profiles, alongside significantly increased antioxidant and antimicrobial activities. These biological activities dose-dependently improved with increasing extract concentrations. This research establishes a foundation for developing novel functional vinegars by leveraging the synergy between sugarcane juice and *C. sappan* wood. Such products expand the range of health-promoting fermented beverages, utilize sustainable and locally available agricultural resources, and possess potent antimicrobial properties, suggesting broader applications in food preservation or therapeutics.

## Figures and Tables

**Table 1:** Total phenolic content (TPC) and antioxidant activity of *C. sappan* wood extract.

Sample	Part of Wood	TPC (mg GAE/g DW)	DPPH Radical Scavenging Activity (mg TE/g DW)	IC_50_ (ppm)	Reference
Stem	Heartwood	44.69 ± 0.72	44.40 ± 0.75	140.00	This study
Stem	Sapwood	4.43 ± 0.08	-	8.50	[37]
	Heartwood	8.25 ± 0.18	-	7.10	
Branch	Sapwood	7.77 ± 0.06	-	24.40	[37]
	Heartwood	4.87 ± 0.12	-	15.60	
Stem	Heartwood	213.12 ± 0.87	-	98.99	[38]

**Table 2:** The ethanol concentration and color of vinegar produced from sugarcane juice supplemented with *C. sappan* extract at 0 g/L (C1), 0.5 g/L (C2), 1.0 g/L (C3), 2.0 g/L (C4), and 4.0 g/L (C5).

Treatments	Ethanol Content (% *v*/*v*)	Color ^1^		
		Lightness	Green–Red Color	Blue–Yellow Color
C1	0.05 ± 0.00 ^d^	94.74 ± 1.39 ^a^	−1.14 ± 0.03 ^a^	12.72 ± 0.79 ^d^
C2	0.09 ± 0.01 ^c^	94.27 ± 1.86 ^a^	−2.09 ± 0.33 ^b^	22.32 ± 0.08 ^cd^
C3	0.15 ± 0.02 ^b^	94.10 ± 0.93 ^a^	−3.00 ± 0.08 ^c^	34.76 ± 8.36 ^c^
C4	0.10 ± 0.00 ^c^	92.88 ± 0.16 ^ab^	−4.64 ± 0.17 ^d^	54.86 ± 0.79 ^b^
C5	0.18 ± 0.01 ^a^	90.09 ± 0.93 ^b^	−4.90 ± 0.59 ^d^	87.72 ± 7.22 ^a^

^1^ Lightness (0, black and 100, white); Green–red color (−, green and +, red); and Blue–yellow color (−, blue and + yellow). Means ± SDs following different letters within a column were significantly different based on Tukey HSD at *p* ≤ 0.05.

**Table 3:** Antimicrobial activity of vinegar produced from sugarcane juice supplemented with *C. sappan* extract at 0 g/L (C1), 0.5 g/L (C2), 1.0 g/L (C3), 2.0 g/L (C4), and 4.0 g/L (C5).

Bacterial Strain	Acetic Acid ^1^	dH_2_O ^2^	Diameter of Inhibition Zone (mm) ^3^				
			C1	C2	C3	C4	C5
*Gram-negative bacteria*							
*Escherichia coli*	26.4 ± 1.1 ^ab^	0	7.6 ± 0.7 ^a^	10.8 ± 0.4 ^a^	14.4 ± 0.8 ^a^	17.0 ± 1.0 ^a^	23.4 ± 1.1 ^a^
*Salmonella enterica*	22.6 ± 0.8 ^c^	0	6.6 ± 0.5 ^a^	8.7 ± 0.4 ^b^	10.7 ± 0.6 ^b^	13.6 ± 0.6 ^b^	16.3 ± 0.5 ^b^
*Enterobacter aerogenes*	23.3 ± 1.2 ^bc^	0	6.9 ± 0.4 ^a^	8.8 ± 0.8 ^b^	10.4 ± 1.2 ^b^	13.4 ± 0.7 ^b^	17.5 ± 1.1 ^b^
*Gram-positive bacteria*							
*Bacillus cereus*	24.1 ± 1.8 ^bc^	0	8.4 ± 1.0 ^a^	10.9 ± 0.6 ^a^	14.5 ± 0.7 ^a^	18.3 ± 0.8 ^a^	22.3 ± 0.7 ^a^
*Staphylococcus aureus*	27.5 ± 0.9 ^a^	0	7.3 ± 0.7 ^a^	9.4 ± 0.9 ^ab^	14.4 ± 1.0 ^a^	18.2 ± 0.7 ^a^	23.3 ± 0.7 ^a^
*Enterococcus faecalis*	22.9 ± 0.8 ^c^	0	8.1 ± 0.6 ^a^	11.2 ± 0.9 ^a^	14.3 ± 0.9 ^a^	18.5 ± 0.7 ^a^	23.1 ± 0.8 ^a^

^1^ Acetic acid (pH 3.0) was used as a positive control. ^2^ Sterile distilled water was used as a negative control. ^3^ Diameter of the inhibition zone included a well diameter of 6 mm. Means ± SDs following different letters within a column were significantly different based on Tukey HSD at *p* ≤ 0.05.

## Data Availability

The original contributions presented in this study are included in the article. Further inquiries can be directed to the corresponding author.

## Supplementary Materials

The following supporting information can be downloaded at: https://www.mdpi.com/article/10.3390/antiox14050590/s1, Table S1: Volatile organic compounds (VOCs) in *C. sappan*-supplemented vinegar products; Table S2: Statistical analysis of the fermentation parameters of sugarcane vinegar products supplemented with *C. sappan* extract at 0 g/L (C1), 0.5 g/L (C2), 1.0 g/L (C3), 2.0 g/L (C4), and 4.0 g/L (C5).
