# Supplementary material for: Enhancing the Bioactive Properties of Sugarcane Vinegar Through Caesalpinia sappan Extract Supplementation: A Novel Approach for Functional Beverage Development
Source: Antioxidants (Basel). 2025 May 14;14(5):590. doi: 10.3390/antiox14050590 (PMC12108335; doi:10.3390/antiox14050590)
Supplement: Supplementary file 1 [file antioxidants-14-00590-s001.zip › antioxidants-3592855-supplementary.pdf]

Table S1. Volatile organic compounds (VOCs) in *C. sappan*-supplemented vinegar products.

VOCs	Name	Rt (min)	% area				
			C1	C2	C3	C4	C5
Esters	Ethyl acetate	5.483	ND	9.317	ND	1.077	0.277
	1-Butanol, 3-methyl acetate	8.341	ND	ND	ND	0.153	ND
	Acetic acid, 2-henylethyl ester	24.929	0.143	0.623	0.710	0.343	0.080
	Dodecanoic acid, ethyl ester	25.578	ND	ND	ND	1.183	0.277
	p-Toluic acid, 4-cyanophenyl ester	26.525	ND	1.080	1.637	2.450	0.980
	2-Ethylhexyl salicylate	30.001	ND	ND	ND	0.653	0.143
	Isopropyl myristate	30.677	ND	ND	ND	0.453	0.117
	Tetradecanoic acid, ethyl ester	31.067	ND	0.017	ND	0.917	0.227
	n-Hexyl salicylate	35.629	ND	ND	ND	0.370	0.080
	Hexadecanoic acid, ethyl ester	36.764	0.263	0.350	0.243	3.973	2.320
	Octadecanoic acid, ethyl ester	42.317	ND	ND	ND	0.787	0.370
	Ethyl oleate	42.863	ND	ND	ND	3.450	1.587
	Oleic acid, ethyl ester		ND	0.140	ND	ND	ND
	Linoleic acid, ethyl ester	44.139	ND	0.153	ND	2.053	1.093
	Linolenic acid, ethyl ester	45.850	ND	ND	ND	0.327	0.327
Carboxylic acids	1-Butanol, 2-methyl	10.268	ND	0.080	0.413	0.483	0.130
	1-Butanol, 3-methyl	10.293	ND	0.357	ND	1.373	0.477
	Acetic acid	15.721	56.073	38.950	59.453	59.340	78.733
	2,3-Butanediol, diacetate	17.601	0.533	ND	ND	ND	ND
	Propanonic acid	17.925	ND	ND	ND	0.020	0.063
	Propanoic acid, 2-methyl	18.652	1.317	1.413	0.733	0.250	0.137
	Butanoic acid	20.047	0.070	0.103	0.070	0.020	0.013
	Butanoic acid, 3-methyl	21.072	3.923	3.627	2.853	0.373	0.153
	α-Terpineol	22.010	0.350	0.190	0.193	ND	ND
	Hexanoic acid	25.417	0.327	0.190	0.293	ND	ND
	Phenol, 2-methoxy	25.940	0.230	0.140	0.743	ND	ND
	3-Hydroxy-2,2,4-trimethylpentyl ester of isobutanoic acid	26.324	0.347	0.480	0.413	0.123	0.047
	2,2-Dimethyl-1-(hydroxy-1-isopropyl) propyl ester of isobutanoic acid	26.911	0.183	0.243	0.207	0.067	0.023
	Heptanoic acid	28.226	0.213	0.160	0.163	ND	ND
	Isolongifolene, 4,5,9,10-dehydro	29.352	ND	ND	ND	0.323	0.097
	2(3H)-Furanone, dihydro-5-pentyl	30.814	1.180	1.573	1.400	ND	ND

	Octanoic acid	31.117	2.157	0.650	1.770	ND	ND
	trans-Edulan	32.257	0.233	0.410	0.273	ND	ND
	cis-Edulan	33.185	0.150	0.200	0.143	ND	ND
	Nonanoic acid	34.049	0.380	0.557	0.727	0.197	ND
	Eugenol	34.181	ND	0.170	0.267	0.117	0.053
	2-Heptadecanone	36.204	0.510	0.183	0.177	ND	ND
	Ar-turmerone	36.818	ND	0.383	0.440	1.303	0.673
	n-Decanoic acid	36.971	0.517	0.357	0.587	0.403	0.083
	Ethyl 9-hexadecanoate	37.462	ND	ND	ND	0.530	0.140
	Phenol, 2,4-bis(1,1-dimethylethyl)	37.927	0.910	1.133	1.533	0.577	0.193
	Dodecanoic acid	42.681	0.987	0.843	0.960	1.567	0.347
	Benzeneacetic acid	44.474	0.777	1.017	0.557	0.423	0.150
	Tetradecanoic acid	48.207	1.573	1.140	1.587	0.613	0.163
	n-Hexadecanoic acid	55.248	7.523	5.977	9.747	4.000	1.543
Alcohols	Ethanol	5.867	ND	18.467	ND	5.033	7.603
	Menthol	20.072	0.063	0.130	0.153	0.167	0.030
	Phenylethyl alcohol	27.423	3.533	6.687	8.820	3.477	1.100
	trans-Nerolidol	30.677	ND	ND	ND	0.300	0.040
Benzene	Benzene, 1,3-bis (1,1-dimethylethyl)	15.108	0.327	0.223	0.173	0.473	0.093
Ketone	Acetoin	12.356	12.963	0.963	1.297	0.087	0.167
Aldehydes	Benzaldehyde	17.843	0.267	0.510	0.603	0.067	ND
	Benzaldehyde, 2,5-dimethyl	25.062	0.963	0.877	0.735	0.200	0.050

Rt, retention time; ND, not detected; %area, % formulated from the area detected of all compounds in vinegar samples; C1, vinegar sample produced from sugarcane juice; C2, vinegar sample produced from sugarcane juice supplemented with 0.5 g/L *C. sappan* extract; C3, vinegar sample produced from sugarcane juice supplemented with 1.0 g/L *C. sappan* extract; C4, vinegar sample produced from sugarcane juice supplemented with 2.0 g/L *C. sappan* extract; C5, vinegar sample produced from sugarcane juice supplemented with 4.0 g/L *C. sappan* extract.

Table S2. Statistical analysis of the fermentation parameters of sugarcane vinegar products supplemented with *C. sappan* extract at 0 g/L (C1), 0.5 g/L (C2), 1.0 g/L (C3), 2.0 g/L (C4), and 4.0 g/L (C5).

Treatments	pH	Total acidity (g/L)	Total sugar (g/L)	Total phenolic content (g/L)
C1	3.03 ± 0.06 [ab]	18.72 ± 0.70 [b]	91.79 ± 0.03 [b]	0.37 ± 0.01 [e]
C2	3.14 ± 0.06 [a]	19.31 ± 0.36 [b]	93.84 ± 0.02 [a]	0.61 ± 0.02 [d]
C3	3.14 ± 0.02 [a]	20.32 ± 0.73 [b]	88.61 ± 0.02 [c]	0.77 ± 0.01 [c]
C4	2.90 ± 0.07 [b]	23.27 ± 0.95 [a]	76.16 ± 0.01 [d]	1.01 ± 0.01 [b]
C5	3.03 ± 0.10 [ab]	22.18 ± 0.35 [a]	69.69 ± 0.01 [e]	2.29 ± 0.05 [a]

Means ± SDs following different letters within a column differed significantly based on Tukey HSD analysis at $p \leq 0.05$.
